# Digestive cancers in Morocco: Fez-Boulemane region

**Published:** 2012-11-02

**Authors:** Laila Chbani, Imane Hafid, Mohamed Berraho, Chakib Nejjari, Afaf Amarti

**Affiliations:** 1 Department of Pathology, Hassan II University Hospital, Fez, Morocco; 2 Department of Epidemiology, Faculty of Medicine and Pharmacy, Fez, Morocco

**Keywords:** Gastrointestinal cancers, colorectal, histology, Morocco

## Abstract

**Introduction:**

To describe the epidemiological and pathological aspects of gastrointestinal cancers in Fez-Boulemane.

**Methods:**

This was a retrospective descriptive study of 1120 gastrointestinal cancers diagnosed between 2004 and 2010 in the department of Pathology of Hassan II University Hospital in Fez Morocco.

**Results:**

The average age of our patients was 53 years with a male predominance in 52% of cases. Digestive cancers in this study are distinguished by the predominance of colorectal and stomach location.

**Conclusion:**

Gastrointestinal cancers are the most frequent cancer in our region. An epidemiological monitoring program is needed.

## Introduction

Digestive cancers are the most common cancers worldwide, with 3 million new cases each year and more than 2 million deaths worldwide each year [1]. Unfortunately, two-thirds of these cases occurred in developing countries. The incidence of digestive cancers shows a wide disparity by sex and geographical area [2]. By their frequency and severity, digestive cancers is considered as a serious public health problem in Morocco; their frequency and evolution continue to be unknown owing to the absence of a national register of digestive cancer. In Fez university Hospital, all cancers diagnosed in the pathology laboratory were prospectively collected in a database so that the bias of clinical registries were largely limited. The aim of this study is to assess the frequency of digestive cancers among all cancers and then in this subgroup to study the epidemiological and histological specificities and compare them to the literature data.

## Methods

The records of 1120 patients with gastrointestinal cancers, who were referred between January 2004 to December 2010 to the Department of Pathology, Hassan II University Hospital of Fez Morocco, were retrospectively analysed. The clinical data of each of these patients were analysed for age, sex, tumor location and histological type. Histological typing of the tumors was based on the World Health Organisation (WHO) digestive tumors classification. All specimens were fixed in formolin liquid 10%, paraffin-embedded and stained with Hematoxylin-Eosin-Safran. When needed, immunohistochemistry using Avidin-Biotin immunoperoxydase method was performed. All statistical analyses were evaluated using the statistical software Epi Info version 2004. Variables were compared by chi-square test. A p value <0.05 was determined significant.

## Results

### Overall findings

We have collected during our study period 1120 cases of gastrointestinal cancer histologically confirmed (20.2% of all cancers) occupying the first row. 620 patients (55%) were men and 500 (45%) were women. The mean age at diagnosis was 55.7±11.5 years old. Among patients, 25% had an age lower than 46.5 years old. 32.7% of patients had an age lower than fifty years old. The most frequent localizations were: colorectum with 41.4%, stomach with 29.6% and hepatobiliary tract with 13.3% (Table 1). The 1120 cases of gastrointestinal cancer were subdivised in 951 carcinomas (85%), 73 lymphomas (6,5%), 24 gastrointestinal stromal tumors (GIST) (2,1%), 10 melanomas (0,8%) and 62 other histological types (5,6%) are mainly represented by liver metastases in 93,5% of cases (Table 2)

**Table 1:** Subsite distribution of 1120 cases of digestive cancer by location

Cancer type	Total N=1120 cases	
	no	%
**Colorectal**	464	41
**stomach**	332	30
**Liver**	91	8
**Small Bowel**	88	8
**Oesophagus**	67	6
**Biliary tract**	58	5
**pancreas**	20	2

**Table 2:** Distribution of cases by histological type

Histological type	No	%
**Carcinoma**	951	84
**Lymphoma**	73	7
**Gastrointestinal stromal tumor**	24	2
**Melanoma**	10	1
**Others**	62	6

### Results by location

**Figure 1:**
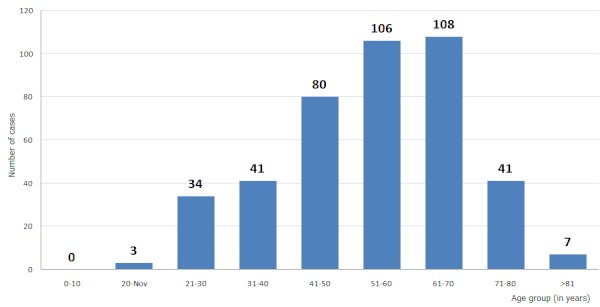
Distribution of colorectal cancer by age

**Colorectum cancer (464 cases):** We identified 464 cases in our series of colorectal cancers, representing 8.30% of all cancers, and occupying the third largest cancers, in terms of frequency. They represent the third male cancer (8%), and the fourth cancer women (7.79%). This is the first digestive cancer and represents 38.82% of digestive cancers. Colorectal cancer is more common among men (232 cases) than in women (203 cases). Statistical analysis of our results gave an average age of 55.32 years, with a median age of 58.5 years. It is increasingly frequent with age, in fact, 75% of cases occur in patients between 45 and 67 years old (Figure 1). The most common histological type was adenocarcinoma (86% cases), some histological types very rare were diagnosed as melanoma (ten cases or 2.1%) and gastrointestinal stromal tumor (GIST) (two cases or 0.4%). The adenocarcinoma was classified, well differentiated in 80.8% of cases.

**Stomach cancer (332 cases):** It represents 29.6% of digestive cancers, and it ranks second place, with an average age of 56 years, sex male is the most affected with 61%. Gastritis with Helicobacter pylori are the main premalignant condition in our series in 68,4%. The majority of cancers were located in antropyloric region and carcinoma represents 83.4% of histological types. Signet ring carcinomas represent 28.5% of epithelial cancer of stomach in our study and are particularly frequent in patient under 50 years old in 45.5% of cases.

**Liver cancer (91 cases):** The 91 cases reported during the study period represent 8.12% of digestive cancers, and 1.64% of all cancers. Recruitment of cases evolved over the years, this is related to the development of techniques of endoscopy and liver biopsies in the department of Gastroenterology in Hassan II Teaching Hospital Fez. The majority of registered cases had occurred among adult patients and elderly ones, since 75% of cases were aged between 50.5 and 70.5 years and 25% of patients were under 50 years. The median age is 59.5 years and average age of 56.89 years. Liver cancers were more frequent in men (53 cases) than women (38 cases) with a sex ratio of 1.39. Liver cancer in our series accounted for 1.83% of all male cancers and 1.46% of all female cancers. 57.14% of registered cases corresponds to secondary sites of biliary-pancreatic, gastric and colorectal tumors. 42.46% of liver cancer cases recorded, 39 cases are primary malignant tumors, the most common is hepatocellular carcinoma, which is 25.27% of the total liver cancer, and 58.97% of primary tumors. It is more common in men (69.57%) than in women (30.43%). However, this frequency is underestimated because liver biopsy is not systematically performed before any hepatic mass and also the diagnosis of these masses is radiological. Moreover, it has been observed particularly in elderly patients, since 75% of patients were between 53.5 and 70 years with a mean age of 59.8 years (p = 0.03). In the second row, we find cholangiocarcinoma, representing 13.19% of all primary cancers of liver, observed mainly in patients aged between 60 and 70.5 years (75% of patients) with an average age of 62.4 years (p = 0.03).

**Small bowel cancer (88 cases):** During our study period, we collected 88 cases of malignant tumors of the small intestine, which account for 1.59% of all cancers and 7.86% of digestive cancers. Analysis of our results by age of the patients showed an average age of 50.48 years with a median age of 54.5 years. 75% of patients were between 43 and 66, and 25% were under 43 years. We noted a male predominance in our series with 60.23% (53 cases) of male cases and 39.77% (35 cases) were female. Cancers of the small intestine accounted for 1.82% of all cancers male and 1.33% of all female cancers. The analysis of cases by histological type showed an adenocarcinoma in 59.09% of cases (52 patients), affecting mainly elderly patients with mean age 56 years (p = 0.0003). Lymphoma in 19.33% of cases (17 patients). Gastrointestinal stromal tumors are noted in 10.23% of cases (9 patients).

**Esophageal cancer (67 cases):** Cancer of the esophagus represented, with 67 cases diagnosed in our series, 5.98% of digestive cancers and 1.21% of all cancers. It is a cancer of elderly patient. Indeed, 75% of patients were aged between 52 and 70. The average age was 58.27 years, while the median age was 61.50 years. The frequency of esophageal cancer is almost equal in women (34 cases recorded, corresponding to 50.75%) and men (33 cases recorded, corresponding to 49.25%) in our series. The epithelial tumors are the most common in our study of which 36 cases (53.73%) are squamous cell carcinomas and 19 cases (28.36%), are adenocarcinomas. In non-epithelial tumors, only one case was recorded, corresponding to a low grade lymphoma. Our statistics show that there is no significant relationship between age of onset and histological type (p = 0.22). Squamous cell carcinoma was more common in women (52.9%), while 57.89% of registered cases of adenocarcinoma, are male. However, we did not find a significant relationship between histological type and sex.

**Cancer of the gallbladder and biliary tract (58 cases):** During our study period, there were 58 cases of cancer gallbladder, which represented 5.18% of digestive cancers and 1.04% of all cancers. 1742 cholecystectomy were recorded during the study period, therefore, 3.32% of them were malignant. The average age of onset was 60.10 years and the median age was 60 years. Moreover, 75% of cases were in patients aged between 54 and 70 years. The extremes of age were 28 and 78 years. 73% of patients were female (corresponding to 42 cases) and 27% only (16 cases) male. Cancer of the gallbladder represented 1.60% of all cancers female and 0.54% of all male cancers. In 96.5% of cases, it is an adenocarcinoma. There was no significant relationship between histological type and age (p = 0.99) nor between histological type and sex (p = 0.99).

**Anal cancer (22 cases):** Anal cancer is a rare cancer in our series, there were only 22 cases representing 1.96% of digestive cancers and 0.39% of all cancers. 75% of cases occurred between 54 and 70 years, while only 25% were noted before the age of 54. The average age of onset was 61.78 years and the median age was 65 years. Anal cancer is more frequent in men who represented 59.09% (14 cases) than women representing 40.91% (8 cases), with a sex ratio 1.75. It represented 0.48% of all male cancers and 0.30% all female cancers. 55.52% of cases are adenocarcinoma. Squamous cell carcinoma take the second place with a rate of 30.43%.

**Pancreatic cancer (20 cases):** Pancreatic cancer is, with 20 cases collected in our study, 1.78% of digestive cancers, and 0.36% of all cancers. This affected mainly adult cancer. Indeed, the average age of onset is 56.65 years and the median age is 55.5 years. 25% of cases were recorded in patients under 49 years old, with a minimum age of 45. 75% of cases occurred in patients aged between 45 and 60, with a maximum of 76 years old. No cases have been recorded in a child or a young adult. The majority of our patients were male, representing 80% of cases (16 cases), while women accounted for only 20% of cases (4 cases). The sex ratio was 4. Pancreatic cancer accounted for 0.54% of all male cancers and only 0.15% of female cancers. 95% were adenocarcinomas.

## Discussion

This study provided a panorama of main cancers of the gastrointestinal tract observed in Fez; northeastern city of Morocco, albeit limited to tissue samples examined by the department of pathology of Hassan II university hospital. Several cases may have been missed because no biopsy or surgical specimen was available. According to various studies, digestive cancers prominently among all cancers [3–5], this series since they joined our rank first of all cancers. According to various studies, men are more affected by digestive cancers than women, with a sex ratio that varies between 1.4 and 2.2. In our series, the sex ratio is 1.24, the average age of onset in our study is similar to that observed in several published studies. By location in the gastrointestinal tract, colorectal cancers were at the first rank [2, 4, 6]. In our series, they occupy the second only to stomach in France, the stomach ranks second with 12.2%, followed by esophageal by 11%, 7% the pancreas, the biliary tract cancer and 2% of the small intestine by 1% of all cases of digestive cancers.

### Study results by location

**Colorectal cancer:** Colorectal cancer is one of the most common cancers worldwide and represents a major cause of cancer death. Approximately one million new cases are diagnosed each year worldwide [7]. In our study, colorectal cancers are the most common in men. In the Maghreb countries, colorectal cancer is the second tract most common in the North of Tunisia in men, and the sixth male cancer with a percentage of 4.5%, while it comes third 68 in women after cancer of the colon and anorectum, and eighth with a percentage of 3.7% [8]. However, according to the Register of southern Tunisia, Colorectal cancers are more common in women, they represent a whole, excluding skin carcinomas, 11.4% of all cancers and are quite common in men (7.8% of all cancers). In Oran, colorectal cancers rank fourth in men and ranking third in women with a frequency of 6.2 and 5.1% respectively [9]. In Egypt, according to the record of the region of Aswan, colon cancer represents 3% of all cancers, and is respectively 3.4% and 2.7% of cancers in men and women [10]. Colon cancer in Rabat is relatively uncommon. It represents the 10th male cancer (2, 6%), and the 17th cancer in women (1.6%). There is in fact, twice as common in men and increases with age. Half the cases occur between 25 and 54, and the average age of patients is somehow higher among women (59.5 years) than men (53.6 years). As for rectal cancer, it is the second in terms of digestive cancer after the frequency of the stomach. The average age of patients was 51.9 in men (median age 54 years) and 49.0 in women (median age 52 years). The register of Casablanca presents similar results, where cancer colon ranks ninth (3.73%) in men and the tenth (2.24%) in 69 the woman [11]. The rectal cancer are followed and held the rank of tenth all male cancers (3.1%) and sixth in women (2.8%) Colorectal cancer is characterized, in our series, with its predominance in young patients. Indeed, 18% of cases occurred in patients under 40 years. Similar results are found nationally. In Rabat, half cases of colorectal cancer occurs between ages 25 and 54. The mean age of patients is slightly higher among women (59.5 years) than men (53.6 years). It is twice as common in men and increases with age. The average age of patients was 51.9 in men (median age 54 years) and 49.0 in women (median age 52 years) [12]. In Casablanca, the average age for women was 57 years for men and 56 years old. For rectal cancer, the average age among women was 57 years and for men 53.4 years old [11]. Further studies are needed to explain the occurrence of this cancer at an early age compared to Western data.

**Stomach cancer:** In our series, cancer of the stomach is second cancer while sex confused or 6.22% of all cancers and 29.6% of digestive cancers. In United States, this cancer accounts for 2.1% of all cancers in men, 1.4% for females and 9.2% digestive cancers for both sexes. According to the Cancer Registry of Rabat Morocco, gastric cancer ranks seventh instead of all cancers (2.8%) and the second cancer tract after colorectal cancer (24.8%). The average age of onset of gastric cancer is 65 in different studies [13]. In our study, it is 56 years but this cancer is particularly frequent in patient under 50 years old in 45.5% of cases. Linitic forms seem to be more frequent in younger patients, and carcinomatosis is much more common at diagnosis. This frequency is certainly not explained by familial forms (only 1.5%) [14] but by other risk factors including environmental (viral infection). The stomach cancer is the second leading cause cancer death after lung cancer, in several countries including France and the United States. At Morocco, it is the fourth leading cause of cancer death.

**Esophageal cancer:** Morocco is characterized by a frequency of esophageal cancer slightly higher rate to Algeria and Tunisia. This cancer is characterized by a male predominantly. In our series, cancer of the esophagus affects almost equally in men and women. The average age of occurrence is variable from one series to another, oscillating about 60 years. In our series it was 58.2 years. The risk factors remain dominated by smoking intoxication [15], Barrett [16, 17] and caustic esophagitis [18], cancer mortality of the esophagus has increased from 1950 to 1976 and strongly then decreased, reaching in 2000 a level below that of 1950. This decrease is mainly due to decrease in alcohol consumption observed since 1950 [19]. Squamous cell carcinoma of the lower third of the esophagus is by far the most common [19], its frequency varies between 71 and 93%, and the analysis of our series shows the frequency of squamous cell carcinoma 53.7%.

**Biliary tract cancer:** Cancers of the extrahepatic bile ducts (EHBD) and gallbladder are rare cancers but not exceptional, their impact has been a slight increase during recent decades [20]. In Morocco, cancer of the gallbladder and EHBD represents 6.1% of digestive cancers. In our series, this location is represented 5.1% of digestive cancers and 1.2% of all cancers. This type of cancer is seen during the sixth and seventh decades, and rarely occurs before the age of 50 years [8]. In gall bladder cancer, the female predominance is one of characteristics that stand out most of all studies epidemiological [20], with a sex ratio of 1.2 to 6.7; in our series, it is 4.5. The most common histologic type is adenocarcinoma in 96.5% which is consistent with the literature data [21].

**Liver cancer:** After metastasis, hepatocellular carcinoma is the primary tumor in terms of frequency. Our results are close to those of countries with low incidence of liver cancer. The United States for example, liver cancer is rare, and represents, with 21,374 cases reported in 2008, 1.5% of all cancers [17]. However, the number of reported cases in our series remains underestimated, since liver biopsy is performed only if the means of exploration radiology (including ultrasound and dynamic imaging) are a vascularization atypical or discordant with the suspected diagnosis [22]. According to the register of southern Tunisia, liver cancer is among men as in women, 1.3% of the overall cancer incidence in the City of Sfax and 1.1% across the region [8].

**Pancreatic cancer:** Pancreatic cancer is the 14th most common cancer in the world, the highest incidence is observed in African American men, the Maori of New Zealand [23]. It is the fifth leading cause of cancer death in both sexes in Western countries. 80 to 90%, the diagnosis is made at a late stage when treatment surgery cannot be radical. In France, it ranks fourth of cancers diagnosed, in our study, pancreatic cancer ranks ninth in gastrointestinal cancer. This type of cancer usually affects the male population over 50 years. In our series, age of onset is 56.8 years, with a sex ratio of 1.3 and about 95% of pancreatic cancers are develop from the exocrine cells (adenocarcinoma ductular).

## Conclusion

Digestive cancers are most common among all cancers of Fez, in contrast to Western data, colorectal and stomach cancer are the most common in our series and predominate among young people. The analysis of sex has objectified a net male and the type of adenocarcinoma which is the most common showing a good correlation with World Series. The increased recruitment of these digestive cancers recent years can be explained by several factors: including rapid population growth, increased of life expectancy, especially the Moroccan's lifestyle that has been changing recently and the implementation of the Hassan II University Hospital, who has improved the level of medicalization of the region. This work can be considered as a first step in the foundation of cancer's registry in the area of Fez and the subsequent national cancer registry.
